# Domestic Summary

**Published:** 1839-04-06

**Authors:** 


### DOMESTIC SUMMARY.

*Annual Interments in the City and County of New York, for the year* 1838: *with accompanying Remarks.* Presented by HENRY G. DUNNEL, City Inspector.

The report presented by Mr. Dunnel, exhibits, under the head of the Diseases of some of the chief systems, the proportionate mortality of the two sexes, at different ages, and of different colours, (black and white.) It is very full, and prepared, evidently, with great care, and, we may suppose, accuracy. The total of deaths, in the city of New York, during the year 1838, was 7533, exclusive of the still-born cases, to the number of 520. The greatest number of deaths for any one month was in August, having been, during that month, 890; and the smallest number 490, in June. Next to these, on their respective sides, were September, which exhibits 798, and May 494.

The proportion of the two sexes and colours, and the rate of deaths are exhibited in the following table:

*The Interments were in the Cemeteries belonging to the following denominations:*

African, 179; Baptist, 165; Catholic, 2685; Dutch Reformed, 316; Friends, 53; German, 141; Hebrew, 25; Marble Cemeteries, 181; Methodist, 1190; Presbyterian, 979; Protestant Episcopal, 625; Potter's Field, 1514.

*From the returns, it appears there died in the following places:—*

The Alms House, Bellevue, 372; Alms House Hospital, Bellevue, 224; Penitentiary Hospital, Bellevue, 47; Penitentiary Hospital, at Blackwell's Island, 34; Small Pox Hospital at Blackwell's Island, 10; City Hospital in Broadway, 121; City Prison, 3; in Westchester County, 63; Long Island, 31; New Jersey, 33.

We shall avail ourselves of the 'Remarks' of Mr. Dunnel, which are a good running commentary on the tabular Report:

"By this report it will be seen that the deaths in 1838 were 679 less than in 1837. Precisely the increase of births in 1837 over those in 1836.

"It may be well, for the gratification of those who have not the time or taste to enter into the investigation, to subjoin a running commentary upon some of the details herein presented.

"There are several interesting results to be gleaned from the precise and peculiar mode of arranging these tables, and which could not be shown by any other method.

"Leaving others to account for the causes, while the facts are simply placed before them, I will premise that, while the total of deaths has been 679 less, the variation in prevalency of different diseases has been immense; from a decreased mortality of 1654 upon some, to an increase of others of 1209.

"The decrease has been chiefly upon the following diseases, viz: of Scarlet Fever, 322; Typhus, 234; Consumption, 233; Convulsions, 178; Measles, 159; Small-Pox, 79; Fever, 74; Teething, 96; Inflammation of the Chest, 40; Diarrhœa, 30; Drunkenness and Delirium Tremens, 31; Child bed, and Puerperal Fever, 24; Dropsy, 19; Bleeding, 12; Mortification, 10; Old Age, 8; and Epilepsy, 5; and 28 less were drowned.

"Of the diseases that have increased, the following stand most conspicuous; of Cholera Infantum, 184. More deaths of this disease occurred this year than ever before, with the single exception of the cholera year, 1834, when it was only 38 greater. In the year 1832, it was 103 less than in this. The increase of Marasmus is 178; Hooping cough, 156; Unknown, 102; Apoplexy, 53; Croup, 31; Remittent Fever, 28; drinking cold water, 20; Malformation, 31; Organic disease of Heart, 18; Bleeding from Lungs, 13; Dropsy of Chest, 13; Scrofula, 12; while of casualties, 12 more occurred, and 8 more were killed or murdered.

The increase of Apoplexy, Unknown, and and drinking cold water, occurred chiefly during the extremely warm part of last summer.

"The number of Still-born and Premature, is precisely the same as last year. There is a curious circumstance connected with this casualty that deserves a remark; that is, the great disproportion of white males to white females, and which does not take place between the sexes of the blacks.

"The greater fatality of male life in the white race, commences before birth, and continues throughout the first year of existence. This year, almost 51 out of every 100, died before reaching 5 years of existence, of whom over 25 were white males, and 22 females—the rest blacks. This inequality does not continue so great after passing the year; there being but trifling variation, (although the males exceed,) between 1 and 2—2 and 5—5 and 10, until between 10 and 20, females predominate; between 20 and 30, they are nearly the same; but between 30 and 50, even to 60, the males are almost double in number to females. Between 60 and 70, they vary a trifle; between 70 and 80, the females outnumber the males, but from 80 upwards, they are equal.

"Throughout the whole series there is a total excess of male deaths, of nearly 10 per cent., and this cannot arise from exposure or casualty alone. There is not a disease of child, except Hooping Cough and Measles, in which the male deaths do not preponderate. The same thing occurs, with few exceptions, at the other periods of life, excluding the peculiar diseases of females, and old age. Of casualties of all kinds, the males exceed females only 148.

"According to the last census, the female population was not 5 per cent. greater than the male. This constant loss of male population (which, taking the whole series embraced in my last year's report, of 32 year's past, has been still greater, having been nearly 12 per cent.) is in some way or another supplied, or, inevitably, the male race would eventually become extinct. It is for the purpose of ascertaining the facts, that a register of births is desirable.

"It is singular, in regard to the deaths of the coloured population, that the males and females differ so little; the coloured females exceed the males only one.

"Of those diseases so fatal under the year, some of them are fatal within a few days of birth. Of Convulsions, 638 died—501 of them under the year; but 159 of them were not 7 days old; between that and 21 days, 177 died; between that and 2 months, 79; and 28 between that and 3 months, leaving but 118 to divide between the remaining three-fourths of the year. Of Malformation and Premature, 77 died under 20 days.

"I have placed in the tables, on a line with the sex and age, the nativity of the persons; in order, if possible, to show the effect, if any, this may have upon disease. By a careful examination of which it will be seen, that of Apoplexy, 49 were natives, and 104 Europeans; of Palsy, Epilepsy and Insanity, one-half of the males were Europeans, and of Bleeding from the Lungs, they exceed the natives. Of Consumption, 1225, there were natives 665, and Europeans 539—11 of adjoining countries, and 10 unknown. The deaths by this disease, excluding casualties of all kinds, is 1 out of 5 of the whole; of which 1 out of 9293 are white natives, 1 out of 4566 blacks, and 1 out of 2877 Europeans. Of Inflammation of the Stomach, 28 were natives, and 36 Europeans. Organic disease of the Heart, 27 natives and 28 Europeans. Of Child-bed and Puerperal Fever, 16 natives to 21 Europeans. Of Intemperance and Delirium Tremens, 40 natives to 55. Suicides, 23 natives to 19. 41 natives and 44 Europeans died of casualties. Out of 22 deaths from drinking cold water, 19 were Europeans; and of Old Age, 57 natives, 54 Europeans, and 3 from the adjoining British provinces.

"I have divided Europe into different sections, in the tables, because of the greater number from some sections; they are all included in these calculations.

"It would tend very materially to an insight into these matters, if the census gave any clue to the proportion of native population of this city; but as it does not, much must be left to conjecture.

"I have made no estimate of the deaths proportioned to the population; because it will necessarily be very unsatisfactory until an accurate register is kept, based upon the deaths, and not upon the interments only, in this city."

---

*Statement of Deaths,—with the Diseases and Ages, in the City and Liberties of Philadelphia, during the year* 1838.

This document is less detailed and less satisfactory than that of New York, just noticed. The alphabetical arrangement of the diseases is pursued, with here and there the cause substituted for the disease, such as Excesssive Heat, Intemperance, Neglect. From such heads as Disease of the Breast, and Disease of the Chest, little information can be obtained. We are aware that these inaccuracies, and loose terminology, are made by the physicians who attended the patients, and who, after death, send in certificates. But something might be done by the medical portion of the Board of Health towards the correction, if not removal of these inaccuracies, by asking the physician for a more definite name of the fatal malady.

The Statement is so far on the side of amalgamation, that it does not designate the proportion of deaths among the blacks to those among the whites; nor even indicate that there are two such different races inhabiting Philadelphia.

The Births and Deaths, in each month of the year 1838, are set forth in the following table:—

The period of the greatest mortality was during the months of July and August, and the least in November. The deaths, within the first year of existence, were 1728; and within the first two years, 2363. The diseases which figure highest in the Report, are, 1st, Consumption of the Lungs, 725; 2d, Summer Complaint, 382; 3d, Convulsions, 302, of which 195 were in children under one year old, 39 in those within the second year, and 33 between the second and fifth years; 4th, Inflammation of the Lungs, 230.

In New York, the cases of Cholera Infantum are 437, those of diarrhœa in children under two years of age, 71. In Philadelphia there were 97 deaths of children under two years of age from diarrhœa. The deaths from Consumption, in New York, were 1225; from Inflammation of the Lungs, 542; from Hooping Cough, 219; and from Croup, 182. In Philadelphia the deaths from Hooping Cough were 27, and Croup, 101. *Bronchitis*, the deaths from which in our city were 118, does not find a place in the New York Report. This omission must be regarded as a defect in the latter.

*Eclectic Journal of Medicine.*

